# Risk analysis and seroprevalence of bovine ephemeral fever virus in Punjab, Pakistan

**DOI:** 10.17221/95/2023-VETMED

**Published:** 2024-03-26

**Authors:** Shahid Nadeem, Rizwan Aslam, Sajjad-ur-Rahman, Muhammad Kasib Khan

**Affiliations:** ^1^Institute of Microbiology, University of Agriculture Faisalabad, Faisalabad, Pakistan; ^2^Department of Parasitology, University of Agriculture Faisalabad, Faisalabad, Pakistan

**Keywords:** enzyme-linked immunosorbent assay, *Ephemerovirus*, lactation, *Rhabdoviridae*, risk factors, vector-borne, virus neutralisation (VN)

## Abstract

Bovine ephemeral fever (BEF) is a vector-borne viral disease caused by the RNA virus which belongs to the genus *Ephemerovirus* and the family *Rhabdoviridae*. To evaluate the effect of the risk factors like the breed of cattle and buffaloes, age, sex, lactation, housing and region on the bovine ephemeral fever virus (BEFV) prevalence, ELISA and virus neutralisation (VN) tests (*n* = 600) were performed for the BEFV prevalence. The seroprevalence in cattle was 45.6% and 42% by ELISA and VN, respectively (*P* = 0.001). The breed-wise seropositive ratio was (55–64%) in cattle and (22.5–18.3%) in buffaloes by VN and ELISA. The sex-wise prevalence was (40–49.4%) in females and (35.8–46%) in males by VN and ELISA in cattle and a similar prevalence was reported in buffaloes. The age-wise prevalence in bovines by ELISA was 5.33, 22.66 and 17.66% in the age group < 1 year, 1–3 years and > 3 years, respectively. The disease prevalence was higher in the age group of 1–3 years. The prevalence was higher during the 3^rd^ lactation in bovines. The region-wise prevalence was higher in the 07 districts while lower (18–21%) in Rawalpindi District by VN and ELISA, respectively (*P* = 0.001). Commercial dairy farms of cattle showed a higher disease prevalence (52% and 44%) than non-commercial farms (38% and 36%) by ELISA and VN, respectively (*P* = 0.227). Exotic cows showed higher disease prevalence (76.67% and 70%) by ELISA and VN. The mortality in bovines was 5% (7.7% and 2.3%) in the cattle and buffaloes. The case fatality of BEFV in bovines was 12.25%. There was a significant effect of the risk factors like the breed, age, sex, lactation, housing and region on the BEFV prevalence. This is the first comprehensive study of BEFV in Pakistan.

Bovine ephemeral fever (BEF) is a vector-borne viral disease caused by the RNA virus which belongs to the genus *Ephemerovirus* and the family *Rhabdoviridae*. Bovine ephemeral fever is prevalent in temperate, tropical, subtropical and other areas of Australia, Asia, Africa, and the Middle East. BEF is of high economic importance for cattle and buffaloes. The major clinical signs include a drop in the total milk production, infertility in male bulls and abortion in females. Recovery might be prolonged, affecting the trade and transportation of infective animals (Walker et al. 2012). Other clinical signs of BEF disease include stiffness, high fever, constipation, lameness, depression and lack of rumination (Quinn et al. 2001). The morbidity rate might be higher (80%) in diseased animals, but the mortality rate is low at 1–2%. The mortality rate is also higher (30%) in fat and healthy animals (Walker et al. 2012). Bovine ephemeral fever virus (BEFV) is transmitted by different vectors like the *Culicoides* species, which consist of biting midges and mosquitoes (Kato et al. 2009).

The epidemics of BEF disease are considered to occur seasonally and to be endemic in Asia, Africa, the Middle East and Australia (Walker and Klement 2015). It was also noted that it is mostly sporadic in hot and humid areas. The temperate areas of the different Asian countries present in the southern region, like Iran, Iraq, Pakistan and Bangladesh, are endemic areas for this disease in buffaloes and cattle (Roya 2008). BEF is commonly subclinical in animals in different hot zones. Outbreaks of disease occur during or after periods of rainfall (Quinn et al. 2011). The BEF virus can survive in winter on vectors. It is a transboundary disease that may spread from one country to another by wind and live animal trading (Yeruham et al. 2007; Zheng and Qiu 2012).

In a survey in Iran, it was found that, in cattle, disease cases were higher in females than males, but no variations were reported according to sex in buffaloes (Momtaz et al. 2012). In Saudi Arabia, a serological survey was conducted in the year 2010 on non-vaccinated cattle which showed higher cases of the disease (24.4%) in male than in female cattle (14.6%) (Zaghawa et al. 2016). In Tibetan yaks, the morbidity rates for BEF are higher in females and premature cattle (Liu et al. 2017).

The treatment of BEF is based on clinical signs and symptoms using non-steroidal anti-inflammatory drugs (NSAIDs) and the infusion of calcium borogluconate in animals having low calcium levels. The disease can be controlled by effective vaccination. Live-attenuated, subunit, inactivated and recombinant vaccines have been used commercially and experimentally. Long-term immunity is produced by live vaccines (Radostits et al. 2006). The disease can be effectively controlled by vaccination of animals before the seasonal onset of the disease and by controlling the vectors for the disease spread (Aziz-Boaron et al. 2012; Aziz-Boaron et al. 2014).

Various studies have been conducted to develop an efficient vaccine for BEF, including live attenuated, inactivated, subunit G protein-based and recombinant vaccines. Two doses of the vaccine administered on days 0 and 21 or three doses administered on days 0, 7 and 36 resulted in 100% protection against the experimental challenge on day 104 (Walker and Klement 2015). Primary vaccination in calves followed by regular boosts with a quality vaccine usually provides satisfactory protection (Lee 2019). In the animals that are commonly seriously affected by BEF having a good body score and are healthy, solid immunity is developed in most of the animals that recover (Quin et al. 2011).

The BEFV diagnosis is based on various serological tests, serum neutralisation, and blocking enzyme-linked immunosorbent assay (ELISA) analysis in which samples were obtained 21 days apart for the detection of the serum conversion. The assay was carried out in a local laboratory without any special equipment (Stram et al. 2005).

Serologically, bovine ephemeral fever was diagnosed by ELISA, by using G1 as the purified recombinant protein as the coating antigen. The reaction time, temperature, and other conditions of ELISA were also optimised (Bakheit et al. 2004). G1-ELISA can be used for screening BEFV in a herd, as it is an inexpensive and rapid disease-detecting tool from serum samples. An ELISA kit could be developed based on G1-ELISA for serological epidemiological studies on a large-scale basis (Balamurugan et al. 2007).

In Pakistan, the BEF disease occurs sporadically. During the summer season, the disease was found in water buffaloes and exotic and local cattle. In Pakistan, the BEF disease was mostly detected based on signs and symptoms (Zahid et al. 2019).

The Punjab province contains 50% of the bovine population of the country. No major study has been reported in Punjab regarding bovine ephemeral fever. BEF is a big problem for the dairy sector in Punjab due to the unavailability of a proper diagnosis procedure and awareness of the disease. Therefore, the present study was designed to determine seromolecular prevalence in bovines and arthropod vectors by using ELISA and VN (virus neutralisation) in eight different regions of the Punjab province in Pakistan. Furthermore, various risk factors were also studied to reveal basic information about the BEF epidemiology.

## MATERIAL AND METHODS

### Geographical location of the study area

Punjab is located at 31.170 406 (31°10'13.46''N) latitude and 72.709 716 (72°42'34.98''E) longitude. Punjab is the largest province population-wise (both humans and animals) in Pakistan. In Punjab, the following eight districts were selected for sample collection, Rawalpindi, Sialkot, Jhelum, Lahore, Mianwali, Faisalabad, Jhang, and Sheikhupura. In Pakistan, the monsoon season starts from June to August, with heavy rainfall throughout the Punjab province.

### Sample collection

A total of 600 dairy animals (*n* = 75/district) showing signs of the clinical disease were collected during 2022. The serum samples (*n* = 300) were each processed for ELISA and VN. The animals were divided into two groups: cattle (*n* = 180) and buffaloes (*n* = 120) for each technique. The risk factors included males and females divided into three age groups: < 1 year, 1 to 3 years, and > 3 years. The mortality and case fatality calculations for the cattle population studied were (*n* = 985) and (*n* = 970) for buffaloes. During this study, a questionnaire was also designed to obtain data for the epidemiological analysis of the risk factors. Blood samples were collected from the jugular veins of the affected animals. The whole blood samples were collected into plain 5 ml K_3_ethylenediaminetetraacetic acid (EDTA) commercially available vacutainers.

### Buffy coat extraction

The blood samples collected from the infected bovines on the 2^nd^ or 3^rd^ day of the disease were centrifuged. Buffy coats were obtained during centrifugation of the unclotted blood at 686 *g* for 5 min in which 20 μl was used for the inoculation.

### Indirect enzyme-linked immunosorbent assay (ELISA)

A Bovine Ephemeral Fever Virus Antibody (Anti-BEFV) ELISA Kit (Abbexa UK Ltd.,Cambridge, UK) was used for the qualitative detection of BEFV in the bovine serum.

The indirect ELISA was performed in 96-well polystyrene microtitration plates. A total of 300 serum samples were collected from the suspected animals during the disease period.

The BEFV antibodies were pre-coated onto a 96-well plate. Controls, test samples, and Horseradish peroxidase (HRP)-conjugated reagents were added to the wells and incubated. According to the ELISA kit protocol, unbound conjugates were removed using wash buffers. A 3,3',5,5'-tetrameth-ylbenzide (TMB) substrate was used to quantify the HRP enzymatic reaction.

After the TMB substrate was added, only the wells that contained sufficient BEFV produced a blue-coloured product, which then changed to yellow after adding the acidic stop solution. The intensity of the yellow colour is proportional to the BEFV amount bound on the plate. The optical density (OD) was measured spectrophotometrically at 450 nm in a microplate reader to determine the presence of BEFV (Zheng and Qui 2012).

### Virus neutralisation (VN) test

A virus neutralisation (VN) test was carried out on a flat-bottom microtitration plate. In the first column, 50 μl of the medium were added, and in the other wells, 25 μl of the medium was added. The serum samples were inactivated at 56 °C for 30 minutes. Then, 25 μl of the serum samples were added to the first column wells. Then two-fold dilutions were made by 25 μl pipetting in each well to the next-last column.

Afterwards, a 25 μl solution was discarded from the last well to make 25 μl of the diluted sample in each well. A 100 TCID_50_ per well virus was added to each well. The virus and serum samples were incubated at 37 °C for one hour to neutralise the virus. Thirty thousand (30 000) Vero cells per well were added to a volume of 150 μl. The plates were incubated at 37 °C with 5% CO_2_ in a moist environment for five days. After five days, the plates were emptied, washed with sodium chloride, and then stained with a crystal violet dye solution prepared in 5% phosphate-buffered saline (PBS) for 30 minutes. The cells were fixed with a 10% formalin solution in an acetic acid buffer. The tests were repeated three times, and the mean was calculated for each test.

The virus neutralisation titre was detected by the highest sample dilution which inhibited 50% of the cytopathogenic effects as compared to the control wells. A titre having a ratio of less than 1 : 4 was considered negative (Almasi and Bakhsheh 2019).

### Reverse transcriptase (RT)-PCR amplification

Seropositive samples were confirmed by reverse transcriptase-polymerase chain reaction (RT-PCR) by using a One-Step RT-PCR kit (Invitrogen, Carlsbad, USA). The sequence of primers used were (5' AGAGCT TGG TGT GAA TAC 3') as the forward primers and (5' CCA ACCTAC AAC AGC AGA TA 3') as the reverse primers. The initial denaturation of the product was performed at 94 °C for 5 min, and denaturation through a total of 35 cycles was performed at 94 °C for 40 s, annealing at 48 °C for 1 min, the primer extension was performed at 72 °C for 40 s and a final extension was performed for 10 min at 72 °C. The PCR products were run on a 1.5% agarose gel and the bands were visualised under ultraviolet light on a gel doc system (El-Habbaa and Radwan 2019).

### Statistical analysis

The difference in the molecular prevalence of the BEF virus according to different biological factors, such as age, species, and sex, was established. The difference was significant at a *P*-value less than 0.05 (Momtaz et al. 2012).

### Epidemiological investigation

The overall seroprevalence (SP) of the BEF virus antibodies was estimated. The SP was defined as the proportion of serum samples in the studied population having antibodies against the BEF virus disease.

Against the BEFV antibodies, the seroprevalence (SP) was calculated by the following formula:

$$\text{SP}=\frac{\begin{array}{c} \text{Total number of positive cases}\\ \text{of disease} \end{array}}{\text{Total number of samples tested}}\times 100$$ (1)

### Mortality rate and case fatality

The mortality rate during the year 2022 in the different districts of disease epidemics was calculated as the ratio of animals that died after showing signs of the BEFV infection. The fatality rate during a year was also calculated as the ratio of animals that died after showing signs of the BEFV infection.

## RESULTS

### Clinical signs and symptoms

Clinical cases of the BEFV disease were selected for the sample collection. The animals showed signs of fever, anorexia, respiratory distress, salivation, nasal discharge, drop in milk production, muscle stiffness, lameness, and recumbence.

### Region-wise prevalence of BEFV by VN

**Figure 1:**
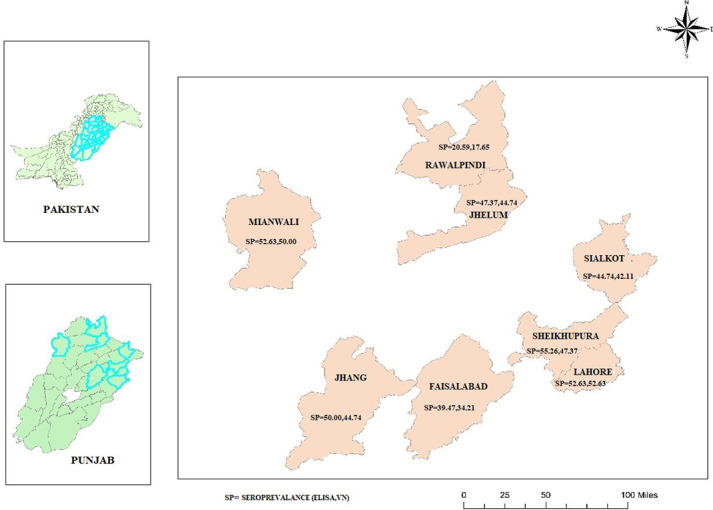
Map of Pakistan depicting seroprevalence in different regions

The disease prevalence was highest at 50, 53, 55, 53, 39, 45, and 47% in Jhang, Lahore, Sheikhupura, Mianwali, Faisalabad, Sialkot, and Jhelum, respectively, while it was lowest at 21% in the Rawalpindi District (*P* = 0.000 1) (Table 1, Figure 1).

**Table 1:** District-wise seroprevalence of BEFV

Tests	ELISA							VN					
Location	total	positive	% age	negative	% age	C.I. (95%)		total	positive	% age	negative	% age	C.I. (95%)
Faisalabad	38	15	39.47	23	60.53	38.19–40.74		38	13	34.21	25	65.79	32.30–36.11
Sheikhupura	38	21	55.26	17	44.74	54.62–55.89		38	18	47.37	20	52.63	47.054 768
Sialkot	38	17	44.74	21	55.26	44.10–45.37		38	16	42.11	22	57.89	41.154 306
Lahore	38	20	52.63	18	47.37	52.31–31.94		38	20	52.63	18	47.37	52.313 194
Mianwali	38	20	52.63	18	47.37	52.31–31.94		38	19	50.00	19	50.00	50.00
Jhelum	38	18	47.37	20	52.63	47.05–47.68		38	17	44.74	21	55.26	44.10–45.37
Jhang	38	19	50.00	19	50.00	50.00		38	17	44.74	21	55.26	44.10–45.37
Rawalpindi	34	7	20.59	27	79.41	16.22–24.96		34	6	17.65	28	82.35	14.28–21.01
													
Total	300	137	45.67	163	54.33	41.53–49.80		300	126	42.00	174	58.00	39.28–44.71
													
*P*-value	0.000 1							0.000 1					

BEFV = bovine ephemeral fever virus; C.I. = confidence interval; ELISA = enzyme-linked immuno sorbent assay; VN = virus neutralisation

### BEFV prevalence based on breed, sex, age, lactation, and housing by ELISA

Antibodies against BEFV were detected in (115/180) in cattle and (22/120) in buffaloes. The SP was 64% (*P* = 0.000 01) and 18.33% (*P* = 0.001 3) in buffaloes and cattle, respectively. The cattle showed a higher number of disease cases.

The BEFV SP was 20, 96.97, and 75% for cattle aged < 1 year, 1–3 years and > 3 years, respectively (*P* = 0.000 01), whereas a similar prevalence was observed at 10, 25 and 20% for buffaloes aged < 1 year, 1–3 years, and > 3 years, respectively (*P* = 0.210). These results indicate more BEF disease cases at the age of 1–3 years in bovines.

**Figure 2:**
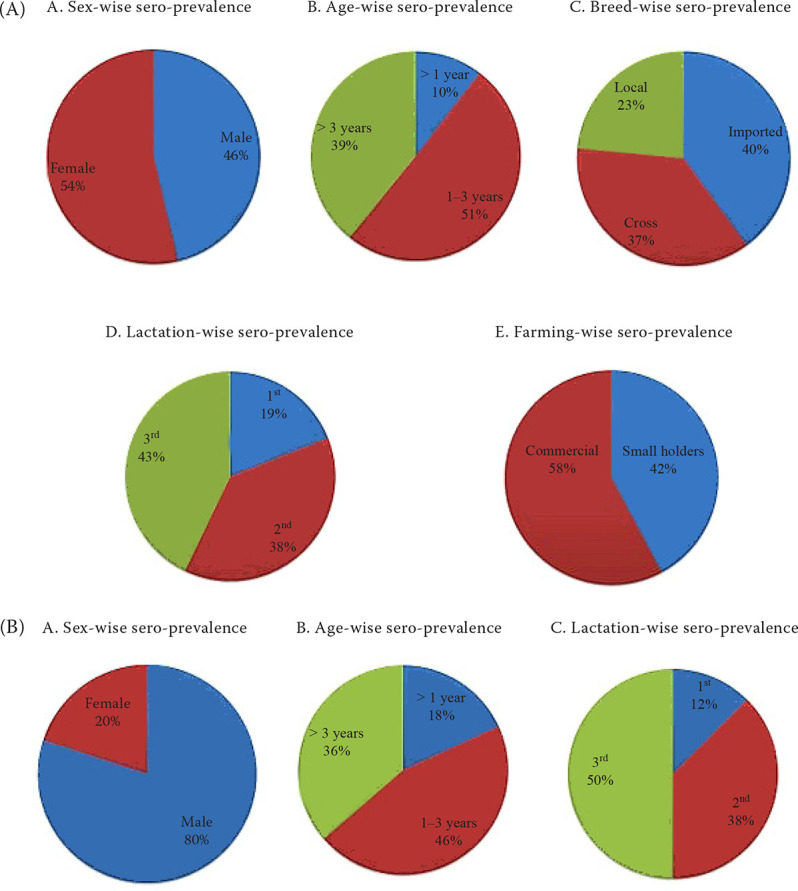
Risk factor based sero-prevalence of BEFV (pie-charts) (A) In cattle. (B) In buffalo

The SP estimates were higher in exotic cattle (76.67%) (95% CI: 76.62–80.72) by ELISA. The SP estimates were higher in the 3^rd^ lactation (60% and 40%) in cattle and buffaloes, respectively, by ELISA, as shown in Table 2 and Figure 2.

**Table 2:** Risk factors of the seroprevalence of BEFV by ELISA and VN

Variable	Categories	ELISA						VN				
		total No. of samples	positive	% age	C.I. (95%)	*P*-value		total No. of samples	positive	% age	C.I. (95%)	*P*-value
**Cattle**												
Sex	total	180	115	63.89	57.17–70.60	0.000 01		180	99	55.00	53.68–56.31	0.171 7
	male	72	42	58.33	56.94–59.71			72	35	48.61	48.37–48.84	
	female	108	73	67.59	63.23–71.94			108	64	59.26	57.37–61.14	
Age	< 1 year	60	12	20.00	15.44–24.55	0.000 01		60	10	16.67	11.60–21.73	0.000 01
	1–3 years	60	58	96.67	89.58–103.75			60	47	78.33	74.02–82.63	
	> 3 years	60	45	75.00	71.20–78.79			60	42	70.00	66.96–73.03	
Breed	imported	60	46	76.67	76.62–80.72	0.000 7		60	42	70.00	66.96–73.03	0.000 3
	cross	60	42	70.00	66.96–73.03			60	36	60.00	58.48–61.51	
	local	60	27	45.00	44.24–45.75			60	21	35.00	32.72–37.27	
Lactation	1^st^	30	08	26.67	24.16–29.17	0.023		30	6	20.00	16.77–23.22	0.015
	2^nd^	30	16	53.33	52.97–53.68			30	14	46.67	49.82–50.17	
	3^rd^	30	18	60.00	58.92–61.07			30	16	53.33	52.97–53.68	
Farming	small holders	50	19	38.00	36.33–39.66	0.227		50	18	36.00	34.06–37.94	0.540
	commercial farms	50	26	52.00	51.72–52.27			50	22	44.00	43.16–44.83	
Variable	Categories	ELISA						VN				
		total No. of samples	positive	% age	C.I. (95%)	*P*-value		total No. of samples	positive	% age	C.I. (95%)	*P*-value
**Buffaloes**												
Sex	total	120	22	18.30	11.50–25.09	0.001 3		120	27	22.50	16.59–28.40	0.266
	male	48	16	33.33	31.06–35.59			48	08	16.67	12.14–21.19	
	female	72	06	8.33	1.40–15.26			72	19	26.39	22.46–30.31	
Age	< 1 year	40	04	10.00	5.04–14.95	0.210		40	05	12.50	7.85–17.14	0.069
	1–3 years	40	10	25.00	21.90–28.09			40	13	32.50	30.33–34.67	
	> 3 years	40	08	20.00	16.28–23.71			40	11	27.50	24.71–30.28	
Lactation	1^st^	20	02	10.00	6.59–13.50	0.091		20	01	5.00	1.05–8.09	0.060
	2^nd^	20	06	30.00	18.56–21.43			20	04	20.00	17.37–22.63	
	3^rd^	20	08	40.00	39.12–40.87			20	07	35.00	33.68–36.31	

BEFV = bovine ephemeral fever virus; C.I. = confidence interval; ELISA = enzyme-linked immuno sorbent assay; VN = virus neutralisation

### Region-wise prevalence of BEFV by VN

A total of 300 serum samples were evaluated by VN from the diseased and recovered bovines. A 1 : 4 titre was observed in the negative serum samples. The district-wise disease prevalence was higher at 45, 53, 47, 50, 34, 42, and 45% in Jhang, Lahore, Sheikhupura, Mianwali, Faisalabad, Sialkot, and Jhelum, respectively, while it was low at 18% in the Rawalpindi District (*P* = 0.000 1) as shown in Table 1 and Figure 1).

### BEFV prevalence based on breed, sex, age, lactation, and housing by VN

A total of 42% of the serum samples were positive for BEFV by the virus neutralisation test. The cattle showed a higher percentage prevalence than the buffaloes, which was 55% and 22.50%, respectively. The BEF-positive samples were evaluated sex-wise in the bovines. In the cattle and buffaloes, females showed a higher number of disease cases (59.25% and 26.38%, respectively). While in the male cattle and buffaloes, the SP was low at 48.61% and 16.66%, respectively.

The SP was 16.67, 78, and 70% for the cattle aged < 1 year, 1–3 years and > 3 years, respectively (*P* = 0.000 01), while there was a similar pattern (12, 32, and 27% for buffaloes aged < 1 year, 1–3 years and > 3 years, respectively) (*P* = 0.069). These results indicate more BEF disease cases at the age of 1–3 years in the bovines.

The SP estimates were higher in exotic cattle (70%; 95% CI: 66.96–73.03) by VN. The SP estimates were higher in the 3^rd^ lactation (53% and 35%) in the cattle and buffaloes by VN, as shown in Table 2 and Figure 2.

### Mortality

Mortality was recorded in the cattle and buffaloes for the BEF disease, and it was found that the mortality percentage in cattle was higher (75/985) at 7.7% than (23/970) in the buffaloes at 2.3%. The overall bovine mortality was 5.0% (Table 3).

**Table 3:** Mortality and case fatality of bovine ephemeral fever virus in the cattle and buffaloes

Variable	Categories	Mortality					Case fatality			
		total animals	deaths	% age	*P*-value		total deaths	sick animals	% age	*P*-value
**Cattle**										
Sex	total	985	75	7.7	0.000 5		75	400	18.75	0.015 6
	male	492	23	4.67			23	185	12.43	
	female	493	52	10.55			52	215	24.19	
Age	< 1 year	328	13	3.96	0.005		13	134	9.70	0.014
	1–3 years	328	35	10.67			35	135	25.93	
	> 3 years	329	27	8.21			27	131	20.61	
**Buffaloes**										
Sex	total	970	23	2.37	0.083		23	400	5.75	0.099
	male	485	6	1.24			6	185	3.24	
	female	485	17	3.51			17	215	7.91	
Age	< 1 year	323	3	0.93	0.098		3	134	2.24	0.114
	1–3 years	323	11	3.41			11	135	8.15	
	> 3 years	324	9	2.78			9	131	6.87	

### Case fatality

The case fatality was studied in the cattle and buffaloes for the BEF disease, and it was found that it was higher in cattle at 18.75% than in buffaloes at 5.75%. The overall case fatality of BEFV in the bovines was 12.25% (Table 3).

## DISCUSSION

In the current study, the prevalence of BEF by the ELISA method was 64.0% in cattle and 18.3% in buffaloes. The cattle showed a higher number of disease cases. The overall prevalence of BEFV in bovines by the ELISA was 45.66%. The positive samples were evaluated according to the sex in the bovines. The cattle and buffalo females showed a higher number of disease cases (67.59% and 22.5%, respectively), and in male cattle and buffaloes, the prevalence was lower (57.5% and 12.5%, respectively). ELISA was used for the BEFV screening in a herd, as it was an inexpensive and rapid disease-detecting tool from the serum samples. The ELISA kit was coated with G-antigen, and the results are comparable to those of other studies.

The BEF disease was diagnosed based on various serological tests, serum neutralisation and a blocking ELISA analysis, in which the samples were obtained 21 days apart for the detection of the serum conversion. The assay was carried out in a local laboratory without any special equipment (Stram et al. 2005).

The overall results of the virus neutralisation tests and blocking ELISA analysis were comparable to each other. The blocking ELISA test has a higher sensitivity and is simpler than the virus neutralisation test; hence, it is a more suitable test for bovine ephemeral fever virus monitoring and diagnosis (Zakrzewski et al. 1992).

Similarly, in the current study, the bovine ephemeral fever virus was detected by different tests like ELISA and VN. The percentage prevalence between these two tests was 45.66% for ELISA and 42% for VN. It was found that the BEFV detection in the suspected diseased cases was higher by the ELISA test method.

Nabukenya et al. (2014) found that the BEFV seroprevalence in Uganda was 7–10% in cattle. These results are low when compared to this current study. In Punjab, the BEFV seroprevalence in cattle by the ELISA and virus neutralisation methods was 64% and 58%, respectively. Momtaz et al. (2012), during a study in Iran, reported the prevalence of BEFV disease at 29% and 17% in cattle and buffaloes, respectively. The seroprevalence of BEFV in female cows was 34.4% to 11.6% in males.

In this current study, the seroprevalence was 64% in cattle and was 18.3% in buffaloes in eight districts of Punjab, Pakistan. The cattle and buffalo females showed a higher number of disease cases (67.59% and 20.8%, respectively). While, in the male cattle and buffaloes, the BEFV seroprevalence was as low as 57.5% and 14.5%, respectively. These results show a similar relationship to the above study, and the BEFV prevalence was higher in the cattle than the buffaloes and was also higher in female bovines.

Similar results were reported in which the seroprevalence of BEFV was 5% to 75%, which varied from county to county, and the species-wise variation was higher (cattle at 65% than in buffaloes at 55%) in Yunnan Province, P.R. China (Lim et al. 2007).

Al-Sultany and Hassan (2013) also observed the same pattern of BEFV disease in bovines, with a higher prevalence (14% in females than in males, which was 10%).

Zaghawa et al. (2016) also reported that the age-wise BEF prevalence in cattle was 20.5, 38.8, and 15.2%, for animals aged < 1 year, 1–3 years, and > 3 years, respectively. These results suggest that the disease prevalence was higher in animals aged 1–3 years. Similar results were found in this current study in which the BEF prevalence was higher in animals aged 1–3 years.

In conclusion, it can be stated that there is a significant effect of the risk analysis on the seroprevalence of the bovine ephemeral fever virus in the different districts of Punjab, Pakistan. The risk factors like age, breed, and sex showed a significant effect on the BEFV seroprevalence. Among different sero-diagnostic techniques for the detection of BEFV antibodies in bovines, ELISA was found to be the most sensitive technique having higher accuracy. This study also showed a higher disease prevalence of BEF among female than male animals. The study also defines the spread of disease in the different districts of Punjab during the monsoon weather due to the high vector population.
